# The Value of the Patient's Criticism

**Published:** 1913-05-17

**Authors:** 


### FROM THE PATIENTS' POINT OF VIEW.

### I.—The Value of the Patient's Criticism.

Patients who have been for some weeks or months inmates of a hospital have usually formed certain impressions and opinions with regard to their experiences while undergoing treatment. These views are sometimes expressed very forcibly; in other cases they are voiced only to personal friends, no matter whether they are favourable or unfavourable. In the latter event, the hospital worker, who may learn much from such verdicts, scarcely gets an opportunity to become acquainted with the patients' point of view. In the case of members of the medical staff and nurses who are daily, and sometimes hourly, occupied with the patients, who can elicit opinions and suggestions by judicious questioning, and who, in their own interests, generally try to appreciate the wishes and anxieties of their patients, the want of such publicity is not so apparent as in the case of the committee-man and the lay worker.

### Importance of Recognising Patient's Wants.

The surgeon, for instance, has learned, through personal experience in hospital and in private practice, to pay attention to those various valuable little hints in regard to comfort and convenience that count for so much in making the after-treatment easy and the subsequent period of convalescence really a restful time. It is his business to know all about these little points. Similarly with regard to the physician who is in charge of a serious case in the medical wards. He, too, knows by experience what are some of the wants of his patient. Unfortunately, in very many cases both physician and surgeon trust largely to the nurse or the sister in charge of the ward. Here, again, when the latter is an experienced individual the patient will have comparatively little to complain about. But the fact remains that there are many little points to which relatively little attention is paid. These are the points which we hope to bring out in this series of papers, the object of which is to give the hospital worker first-hand opinions, emanating from men and women who have actually been in-patients of a hospital, and who have endeavoured to put in writing their impressions with regard to their treatment.

We feel sure that such a series will be helpful and illuminative to hospital workers. Nothing is gained by suppressing real grievances through an exaggerated sense of gratitude for the undoubtedly great help that has been given in assisting the patient to tide over a severe complaint. On the contrary, the patient who tells the hospital authorities frankly where and how, in his opinion, they have erred, deserves our thanks, for by so doing he may often succeed in removing undoubted sources of complaint which, had attention not been drawn to them, might have remained to vex other patients in the future. Suggestions based on personal experience are always valuable, and, therefore, always to be welcomed. It may be, of course, that many of these suggestions owe their being to ignorance of what may be called hospital routine, or to a want of familiarity with the exigencies of medical and surgical treatment. In that case it would be easy to point out that the grievances, if grievances they are, are, in present circumstances, unavoidable. But there are certainly suggestions that stand on a different footing and that are the outcome of mature reflection on certain points which have specially appealed to patients. It is this kind of suggestion that we invite, and we appeal to readers who have been patients in hospitals, and who have considered suggestions to make, to let us have their views. We shall welcome any expression of opinion that is based on actual experience, and any criticism that is knowledgeable, suggestive, or thoughtful, for we consider that the exposition of such views, so long as they are the real personal opinion of people who have actually been in a position to criticise, must be of help to hospital workers.

There are certain necessary qualifications for this task of acting the critic to which it may not be out of place to refer here. In the first place, the critic must have been an inmate of a hospital or of a nursing home attached to a hospital. With the vagaries of treatment in private nursing homes we can scarcely be expected to deal, but matters that concern individual hospitals have also an interest for workers in other institutions. In the second place, it is worth while to say under what conditions and in what particular circumstances the critic came to be in hospital. It is obvious that with the variety of treatment accorded, what may be a just cause for complaint in one instance might be quite a trifling mistake in another. Thus a patient who has been operated upon for a grave gastric or intestinal complaint, in which certain restrictions as to diet are necessary in the after-treatment, cannot honestly complain about this limitation of diet, while on the other hand one whose condition does not necessitate such rigid restriction might justifiably object to undue restriction, semi-starvation, and improper food.

It is, therefore, necessary to know under what conditions the patient was in hospital, in order to be able, to some extent at least, to test the soundness of the views expressed and the practicability of the suggestions made. Our first article deals with the personal experiences of a medical man, some time ago an inmate in one of our hospitals as the result of an attack of appendicitis, upon whom the intermediate operation was performed. Our contributor has had sufficient hospital experience to make his suggestions and views worth considering by hospital workers. Future articles in the series will deal with the experience of lay patients, as well as of patients who have had similar experience of hospital routine, and we cordially invite readers who have suggestions to make, or views to express in regard to their own cases, to take part in the discussion.

